# Effectiveness and tolerability of lactic acid vaginal gel compared to oral metronidazole in the treatment of acute symptomatic bacterial vaginosis: a multicenter, randomized-controlled, head-to-head pilot study

**DOI:** 10.1186/s12905-024-03513-1

**Published:** 2025-01-06

**Authors:** Fiona Tidbury, Grégory Brülhart, Gabriela Müller, Elena Pavicic, Susanna Weidlinger, Gerrit Eichner, Michael von Wolff, Petra Stute

**Affiliations:** 1 Department of Obstetrics and Gynecology, University Clinic of Bern, Friedbuehlstrasse 19, Bern, 3010 Switzerland; 2 Private Practice for Gynecology, Place de la Gare 15, Fribourg, 1700 Switzerland; 3 Mathematical Institute, Justus-Liebig University of Giessen, Arndtstraße 2, 35392 Giessen, Germany

**Keywords:** Bacterial vaginosis, Metronidazole, Randomized controlled trials, Lactic acid, Vaginal microbiome

## Abstract

**Background:**

Bacterial vaginosis (BV) is a prevalent vaginal condition among reproductive-age women, characterized by off-white, thin vaginal discharge with a fishy odor. It increases susceptibility to sexually transmitted diseases (STDs) and pelvic inflammatory disease (PID). BV involves a shift in vaginal microbiota, with reduced lactobacilli and increased anaerobic bacteria. Standard treatment with oral metronidazole has been shown to have a limited long-term efficacy, possibly due to biofilm persistence. Alternative treatments, such as lactic acid vaginal gel, aim to restore vaginal pH and lactobacilli. This pilot study compares the efficacy and tolerability of lactic acid gel to standard oral metronidazole for acute BV treatment in non-pregnant women.

**Methods:**

A total of 32 women with acute BV were recruited and assigned to either the treatment group (*n* = 16) where they applied a lactic acid vaginal gel for 12 days, or the control group (*n* = 16) which received 500 mg oral metronidazole twice daily for seven days. A number of objective and subjective parameters including the Amsel score, the Nugent score and a subjective symptom score were recorded at day 0, three weeks, three months, and six months after the study start.

**Results:**

In the short-term, lactic acid vaginal gel showed inferior clinical (Amsel criteria) and microbiological (Nugent score) cure rates compared to metronidazole. However, it performed equally well regarding subjective symptom improvement and BV recurrence prevention after up to six months.

**Conclusion:**

Lactic acid vaginal gel was generally very well tolerated and showed mixed but promising results as a stand-alone treatment for acute BV.

**Trial registration number:**

NCT02042287 (22.01.2014).

**Supplementary Information:**

The online version contains supplementary material available at 10.1186/s12905-024-03513-1.

## Introduction

Bacterial vaginosis (BV) is one of the most common vaginal complaints among women of reproductive age, with the most common symptoms being vaginal discharge that is off-white, thin, and has a fishy smell [1]. Left untreated, BV can make women more susceptible to acquiring sexually transmitted diseases (STD) including gonorrhea, chlamydia [2], genital herpes, and HIV, which can lead to pelvic inflammatory disease (PID) [3].

The healthy vaginal microbiome is predominantly inhabited by a variety of Lactobacillus species, which constitute 90–95% of the total bacteria. They produce various metabolites such as lactic acid, bacteriocins and hydrogen peroxide (H_2_O_2_), which maintain the vaginal pH ≤ 4.5 (range 3.8–4.4) and therefore create an unfavorable environment for pathogenic microorganisms [4]. In BV, the concentration of H_2_O_2_-producing lactobacilli is reduced and other species become more prevalent, contributing to the characteristic symptoms of BV [5]. These anaerobes raise the pH and produce amines that increase vaginal transudation and squamous epithelial cell exfoliation, leading to the typical discharge and malodor [1].

The mechanism behind this vaginal dysbiosis is unclear; however, a number of risk factors have been associated with BV. These include hormonal changes, medications (e.g. antibiotics, immunosuppressants), foreign bodies, excessive personal hygiene (e.g. vaginal douching), number and frequency of sexual contacts, smoking, HIV infection, black ethnicity, low socioeconomic status and many more [4]. Recent research suggests that uterine cervical intraepithelial abnormalities and cancer may also be influenced by molecular disturbances such as oxidative stress and reactive oxygen species (ROS) in the vaginal space, highlighting the need to consider broad biological factors [6].

BV is generally diagnosed using either the Amsel criteria or the Nugent score. The most widely adopted diagnostic criteria in clinical settings are the Amsel criteria, since they are the most accessible, fast, and affordable method. Alternatively, the Nugent scoring system (NSS) can be used to diagnose BV, where gram-stained vaginal smears are examined microscopically and given a score between 0 and 10. The Nugent score allows for a more gradated evaluation of vaginal flora, is less dependent on the clinician and shows a higher sensitivity than the Amsel criteria [4].

According to current guidelines, first-line treatment of BV is oral metronidazole, 500 mg twice a day for seven days. Alternatives include a metronidazole intravaginal gel or clindamycin cream/ovules for vaginal, tablets for oral application [7]. Both vaginal and oral application show similar cure rates of 80–90% after four weeks. However, these current treatment methods have not been proven to be sufficiently efficient in the long-term, with recurrence rates of up to 69% twelve months after treatment [4]. It has been hypothesized that this is due to the production and persistence of the biofilm (consisting mainly of the bacteria *Gardnerella vaginalis*) despite successful initial antibiotic treatment [8].

An alternative approach to treatment involves using a lactic acid vaginal gel to lower vaginal pH, which supports the recolonization of lactobacilli. The low vaginal pH contributes to the lysis of exfoliated epithelial cells and release of glycogen into the vaginal lumen to be metabolized by lactobacilli [9]. There is evidence supporting the in vitro use of lactic acid to inactivate BV-associated bacteria without affecting vaginal lactobacilli [10]. A recent systematic review by Plummer et al. compared current studies examining the in vivo use of vaginal lactic acid and found mixed results, the vaginal lactic acid product performing equally or significantly inferior to metronidazole. These studies differed greatly in design and had a medium to high risk of bias [11]. We conducted a randomized controlled two-armed pilot study comparing the effect of a lactic acid vaginal gel (Gynofit^®^) containing the active ingredients lactic acid and glycogen to standard oral metronidazole in the treatment of acute BV.

## Materials and methods

### Study population

Patients were recruited for the study at the Department of Obstetrics and Gynecology of the University Hospital Bern, and at the practice of Dr. med. Grégory Brühlhart and Dr. med. Gabriela Müller in Fribourg, Switzerland, between May 2014 and October 2019 and were screened for eligibility. This study was approved by the Cantonal Ethics Committee Bern (No. 2016_01992) and was reported according to the CONSORT guidelines (Supplementary file 1).

Inclusion criteria were:

- Age: Women above 18 years of age were eligible to ensure adult participants who could provide informed consent.
- Informed Consent: Only women who provided signed informed consent were included, ensuring that participants understood the study and agreed to participate.
- Symptomatic BV: Participants needed to present with acute symptomatic bacterial vaginosis (BV), diagnosed using the Amsel criteria. This includes increased homogeneous vaginal discharge, vaginal pH > 4.5, a positive whiff test (characteristic amine odor), and the presence of clue cells on a wet mount. These criteria ensure that only those with clinically confirmed BV were included.

Exclusion criteria were:

- Language Proficiency: Women with insufficient knowledge of German or who were illiterate were excluded to ensure they could fully understand study procedures and requirements.
- Pregnancy: Pregnant women were excluded to avoid any risk to the fetus and because pregnancy can influence vaginal microbiota and BV symptoms.
- Other Acute Illnesses: Participants with other acute illnesses were excluded to avoid confounding factors that could affect study results.
- Allergies: Women with known allergies to any of the investigational product ingredients were excluded to prevent adverse reactions.
- Other Infections: If laboratory investigations revealed other infections, such as Chlamydia trachomatis, Trichomonas vaginalis, or Neisseria gonorrhoeae, the subjects were withdrawn from the study. This was done to ensure the study focused solely on BV and to provide appropriate treatment for these infections.

### Study design

At the study start (visit 1), a detailed general medical and gynecological history was taken (Supplementary file 2). Any current symptoms were quantified using a subjective symptom score, assigning one point per symptom and a maximum total of six points according to the following perceived symptoms: (1) vaginal discharge, (2) vaginal odor, (3) vaginal pain, (4) vaginal itching, (5) burning sensation during urination and (6) vaginal dryness.

A urine sample was given to exclude pregnancy and examined for elevated leukocytes, nitrite, glucose, erythrocytes and protein using a urine test strip. The patients then underwent a vaginal speculum examination by a clinician including a vaginal swab which was used for the assessment of the Amsel criteria. A diagnosis of BV was made if three of the four criteria were fulfilled: (1) increased homogeneous thin gray/white vaginal discharge, (2) elevated vaginal pH > 4.5, (3) characteristic amine (fishy) odor of the vaginal discharge after alkalization with 10% potassium hydroxide (KOH) solution, and (4) presence of clue cells on wet mount.

#### Microscopy

Vaginal swabs were sent to the Institute for Infectious Diseases of the University of Bern, where they were gram-stained and assessed for BV based on the relative concentration of gram-positive rods (Lactobacillus), gram-negative and gram-variable rods or cocci (Gardnerella/Bacteroides), and curved gram-negative rods (Mobiluncus) under 100x magnification, resulting in a Nugent score between 0 and 10. A Nugent score of < 3 was interpreted as BV negative, an intermediate score of 4–6 was registered as BV positive if there were clue cells present, and a Nugent score of 7–10 was regarded as BV positive, irrespective of the presence of clue cells.

#### Bacteriology

Further vaginal and cervical swabs were cultured for a total of 72 h for *Neisseria gonorrhoeae*,* Mycoplasma hominis*,* Ureaplasma urealyticum* and *Gardnerella vaginalis* at the Institute for Infectious Diseases of the University of Bern, and the presence of *Chlamydia trachomatis* was ruled out by means of nucleic acid amplification (PCR).

#### Randomization and Treatment

After baseline assessments and screening for eligibility, randomization to either of the two treatment arms was performed based on a pre-defined, computer-generated block randomization list at the Department of Obstetrics and Gynecology of the University Hospital Bern to ensure equal sample numbers. The allocation was concealed from the study staff to prevent any potential influence on the treatment assignment. Specifically, the randomization list was generated and maintained separately from the clinical staff, and the allocation process was carried out by an independent third party. This means that the staff involved in recruiting and treating participants were unaware of the allocation sequence until the point of randomization, ensuring that treatment assignment was conducted without bias. The study employed a parallel-group design, and the participants were allocated to either the lactic acid vaginal gel (Gynofit^®^, intervention) or metronidazole (control) group, each study group consisting of 50% of the total subjects respectively (16/32). All the pilot study products were provided by Tentan AG and were labelled in accordance with local study site regulations for investigational products. All kit boxes were labelled with the randomization number and stored in a locked area at a temperature between 4 and 25 °C, where the temperature was logged by site personnel once weekly. Study group 1 received the trial product, Gynofit^®^, an acidifying gel containing lactic acid and glycogen as the active ingredients, and excipients composed of water, propylene glycol, hydroxypropyl methylcellulose, caprylyl glycol, levulinic acid, sodium levulinate, sodium hydroxide, sodium lactate, glycerin and p-anisic acid. Indication and dosage were in congruence with the authorization by the Swiss Agency for Therapeutic Products (registered medical device, registration number 10-355-717, licensed 12.08.2010). The patients were instructed to apply one single-use applicator vaginally containing 5 ml daily for a total of 12 consecutive days. Study group 2 received standard antibiotic treatment, oral metronidazole 500 mg twice daily for a total of seven days. Due to different treatment durations and product applications, the patients and clinicians remained nonmasked over the course of the trial.

#### Adherence measurement

Adherence to the study treatment was assessed by multiple methods: Direct Observation: For the gel group, adherence was monitored through direct observation of applicator usage during follow-up visits. Patients were asked to return any unused applicators and were questioned about their adherence to the prescribed regimen. This direct feedback helped ensure accurate adherence reporting. Patient Diaries: Patients in both groups were instructed to maintain a daily diary documenting each dose of their respective treatment. These diaries were reviewed during follow-up visits to verify adherence. Phone Interviews: Periodic phone interviews were conducted to reinforce adherence and address any issues or concerns patients might have had regarding the treatment regimen. This method provided additional support and opportunity for adherence counseling.

### Follow-up

Each woman was seen three weeks after start of treatment (visit 2). During these visits a vaginal speculum examination was performed to determine the Amsel Score, and vaginal and cervical swabs were taken for the analysis of the Nugent score and cultures of *Gardnerella vaginalis*,* Trichomonas vaginalis*,* Mycoplasma hominis*,* Neisseria gonorrhea*,* Ureaplasma urealyticum* and *Chlamydia trachomatis* as done at the study start. Furthermore, the subjective symptom score was recorded in the form of a questionnaire.

Visits 3 and 4 took place at three and six months after study start and involved a telephone follow-up call, where the patients were questioned on various parameters: the onset of new diseases, new medication, pregnancy, newly occurring genital infections such as BV, chlamydia, gonorrhea, trichomoniasis, syphilis and genital herpes. They were also required to describe their current symptoms, which resulted in a subjective symptom score. At every follow-up visit, patients were questioned about the perceived efficacy and tolerability of their treatment, and whether or not they would recommend it.

### Statistical analysis

Based on expected success rates for metronidazole of p(M) = 0.9 and for Gynofit^®^ of p(G) = 0.8, and a type I error of α = 0.05 a power analysis for a two parallel-sample proportions procedure of non-inferiority of Gynofit^®^ against metronidazole was run to estimate the necessary sample size required for a test power of 0.80. The null (inferiority) hypothesis was H_0_: p(G) ≤ p(M) - δ and the alternative (non-inferiority) hypothesis H_1_: p(G) > p(M) - δ with a non-inferiority margin of δ > 0. This means that H_1_ describes an expected success rate for Gynofit^®^ which is not much less (limited by δ) than that for metronidazole, and H_0_ describes the inferiority of Gynofit^®^ to metronidazole of at least δ. A non-inferiority margin of δ = 0.3 was chosen and yielded a total of 14 patients per group required for a study power of 0.90. Accounting for potential drop-outs of 10% the final sample size was increased to 16 per group (32 patients in total).

For statistical analyses and graphics, we used R version 4.2.1 [12] together with the R-package lattice [13]. Cross-sectional comparisons between the two randomization groups were done with Student’s two-sample t-test for variables considered to be normally distributed. Diagnostic normal quantile-quantile-plots using the R-package car [14] were used to check the normality assumption. Student’s test was appropriate because there was no evidence to assume different variances between the two groups. For success proportions, cross-sectional comparisons between the two randomization groups were done with Fisher’s exact test because of the small sample sizes. We analyzed the longitudinal data of the ordinal symptom score by means of a non-parametric method using ANOVA-type tests [15]. The procedure had been implemented in R version 4.2.1 and validated on corresponding designs [16]. To correct for multiple testing, if necessary or sensible, p-values were adjusted by the Holm-method. The statistical significance level was 0.05. All the data from all visits were used for the statistical analysis, despite some women dropping out before many of the follow up visits.

## Results

### Baseline characteristics

A total of 32 women were included in the study. Table 1 shows the distribution of the baseline characteristics between the two treatment arms, where no significant difference was found (*p* > 0.05). All of the women were in good general condition with no acute illnesses, some of whom, however, had chronic health conditions such as arterial hypertension (*n* = 4), endometriosis (*n* = 1), hypothyroidism (*n* = 1), alopecia (*n* = 1), and acne vulgaris (*n* = 2). Our microbiological findings also showed no significant differences between the two treatment arms in the prevalence of *Gardnerella vaginalis*,* Mycoplasma hominis*, and *Ureaplasma urealyticum*. All women presented an Amsel score of ≥ 3, the mean being 3.44 (std. dev. = 0.73) in the vaginal lactic acid group and 3.8 (std. dev. = 0.56) in the metronidazole group. On microscopy, the vaginal lactic acid group showed a BV prevalence of 9 in 16 (56.25%) and the metronidazole group a BV prevalence of 8 in 16 (50%) based on Nugent score and the presence of clue cells. In each group, both the Nugent score and the clue cells assessment were missing for one woman, i.e., the two rates may have been even 10 and 9 in 16, respectively.

**Table 1:** Baseline patient characteristics

	study group 1 (lactic acid vaginal gel)		study group 2 (metronidazole)	
	*n* = 16		*n* = 16	
*Basic characteristics*				
Age (years)^1^	46.4 (11.0; 29–62)		37.9 (12.7; 21–65)	
BMI (kg/m²)^1^	23.3 (2.7; 20–29)		25.0 (6.8; 19–42.3)	
no. vaginal births (n)^1^	1.5 (1.4; 0–4)		0.75 (1.1; 0–3)	
	n	%	N	%
Sexual activity	14	87.50	13	81.25
Smoking	4	25.00	7	43.75
Alcohol consumption > 1x/week	3	18.75	3	18.75
Hormonal contraception (combined oral contraceptive pill, progestin-only pill, LNG-IUD, vaginal contraceptive ring, implant, injection, contraceptive patch)	4	25.00	5	31.25
*Microbiology*				
Gardnerella vaginalis	10	62.50	11	68.75
Mycoplasma hominis	2	12.50	3	18.75
Ureaplasma urealyticum	7	43.75	8	50.00

^1^ given as mean (standard deviation; minimum – maximum)

Abbreviations: BMI = Body Mass Index, LNG-IUD = Levonorgestrel releasing intrauterin device

### Objective outcomes

Table 2 presents the objective study outcomes. The primary outcome was the cure rate based on Amsel criteria. When comparing the Amsel criteria between visit 2 (three weeks) and baseline (visit 1), the metronidazole group with a cure rate of 14 in 14 (100%) performed significantly better than the lactic acid vaginal gel group with 6 in 10 (60%) (*p* = 0.020). However, when the cure rate was based on the Nugent score, there were no significant group differences (NS < 7: 5 of 8 (62.5%) in the lactic acid vaginal gel group vs. 11 of 11 (100%) in the metronidazole group). If the NS was combined with the criterion clue cells (any intermediate flora with an NS of 4–6 was only interpreted as BV-negative if there were no clue cells present) the metronidazole group again performed significantly better than the lactic acid vaginal gel group (*p* = 0.024).

At three months (visit 3) and six months (visit 4), patients were asked whether or not they had been diagnosed with recurrence of BV since the initial treatment. After three months (visit 3), this was true for 4 of 13 (30.8%) patients in the lactic acid vaginal gel group and 2 of 14 (14.3%) in the metronidazole group (*p* = 0.385). The respective numbers after six months (visit 4) were 3 of 8 (37.5%) and 1 of 7 (14.3%) (*p* = 0.569).

To examine the efficacy of lactic acid vaginal gel in patients with certain risk factors, the odds ratio was determined for the cure rate based on Amsel criteria at week three. These odds ratios were calculated for smoking (OR = 0.00; *p* = 0.03), hormonal contraception (OR = 0.63; *p* = 1), and testing positive for *Gardnerella vaginalis* (OR = 1.58; *p* = 1).

**Table 2:** Objective outcomes

	study group 1 (lactic acid vaginal gel)		study group 2 (metronidazole)		Fisher’s Exact Test
*Cure rate according to Amsel criteria* ^*1*^					
visit 2 (3 weeks)	6/10 (60%)		14/14 (100%)		*p* = 0.020
*Mean Amsel score difference versus study start*					
visit 2 (3 weeks)	-1.60		-2.77		*p* = 0.013
*Cure rate according to Nugent score* ^*2*^					
visit 2 (3 weeks)	5/8 (62.5%)		11/11 (100%)		*p* = 0.058
*Cure rate according to Nugent score and clue cells* ^*3*^					
visit 2 (3 weeks)	4/10 (40%)		10/11 (90.9%)		*p* = 0.024
*Recurrence rate* ^*4*^					
visit 3 (3 months)	4/13 (30.8%)		2/14 (14.3%)		*p* = 0.385
visit 4 (6 months)	3/8 (37.5%)		1/7 (14.3%)		*p* = 0.569

^1^ cure defined as: less than 3 of 4 Amsel criteria for BV (homogeneous discharge, pH > 4.5, amine test, clue cells)

^2^ cure defined as: Nugent score < 7

^3^ cure defined as: Nugent score < 7 and lack of clue cells on microscopy

^4^ telephone follow-up, BV diagnosed by clinician

### Subjective outcomes

At every visit, patients were required to self-report on their subjective symptoms, resulting in a symptom score ranging from 0 to 6. These results are presented in Table 3.

**Table 3:** Subjective outcomes

	study group 1 (lactic acid vaginal gel)			study group 2 (metronidazole)		Welch’s or Students t-test or Fisher’s exact test
*Mean symptom score difference versus visit 1*						
visit 2 (3 weeks)	-1.85		-2.08			*p* = 0.715
visit 3 (3 months)	-2.08		-2.23			*p* = 0.787
visit 4 (6 months)	-1.88		-2.29			*p* = 0.609
*Complete symptom relief* ^*1*^						
visit 2 (3 weeks)	8/14 (57.1%)		9/14 (64.3%)			*p* = 1.000
visit 3 (3 months)	9/13 (69.2%)		10/14 (71.4%)			*p* = 1.000
visit 4 (6 months)	5/8 (62.5%)		6/7 (85.7%)			*p* = 0.569

^1^ complete symptom relief defined as: symptom score = 0

**Fig. 1:**
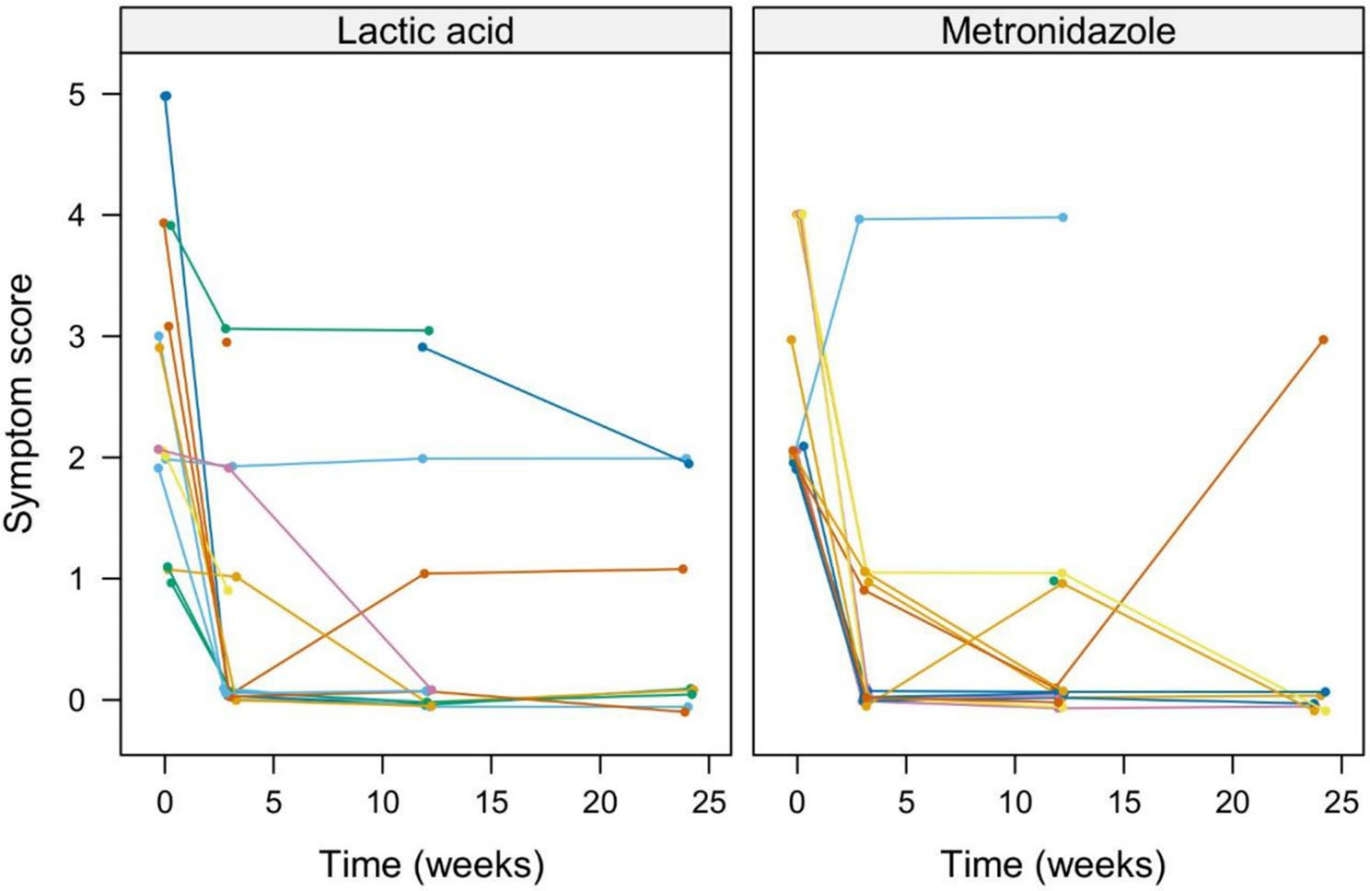
Change in symptom score. Non-parametric repeated measures ANOVA-type test: *p* = 0.817 for the interaction between time and treatment

The most prevalent symptom at study start was vaginal discharge at 71%, followed by vaginal odor, itching, dryness, burning sensation, and pain at 69%, 31%, 28%, 22% and 12%, respectively. The mean symptom score reduction showed no significant differences between groups at all visits. We conducted a non-parametric analysis for longitudinal data (Brunner & Langer Test, ANOVA-type) which also showed no significant differences in the symptom score curves (Fig. 1). At the end of the study at month 6 (visit 4), 62.5% in the lactic acid vaginal gel group and 85.7% in the metronidazole group were completely free from symptoms. This is due to four symptomatic women in total experiencing a BV recurrence at the time of follow-up. All of the recurrence-free women reported having no symptoms at six months.

### Follow-up and drop-out

A total of five women were lost to follow-up by visit 3, and by visit 4 only 15 out of the 32 women were available for the telephone questionnaire, as shown in Table 4. The reasons for cessation were unclear; however, the rate of drop-out remained equal between both arms. By visit 4, five patients in the lactic acid vaginal gel group had received antibiotic treatment due to recurrence of BV. This was also the case for two women in the metronidazole group. These were also the same women who reported a symptom score of > 0 at visit 4. None of the patients tested positive for *Chlamydia trachomatis*,* Neisseria gonorrhea* or *Trichomonas vaginalis* at any point in the study.

**Table 4:** Follow-up and drop-out

	study group 1 (lactic acid vaginal gel)	study group 2 (metronidazole)
visit 1 (study start)	16	16
visit 2 (3 weeks)	13	14
visit 3 (3 months)	13	14
visit 4 (6 months)	8	7

### Adverse events, tolerability, compliance

At visit 2, patients were required to bring the empty applicators and blisters to ensure compliance. All adverse events were recorded in the patient dossiers. Headache (*n* = 1), nausea (*n* = 2), ageusia (*n* = 1), and itching (*n* = 1) were all reported in the metronidazole group, but no adverse events were reported in the lactic acid vaginal gel group. None of the adverse events were severe or caused any of the patients to cease participation in the study. Additionally, the lactic acid vaginal gel group was questioned on the subjective efficacy and tolerability of the treatment (1 = very good, 2 = good, 3 = satisfactory, 4 = unsatisfactory). The metronidazole group was not questioned. The mean subjective efficacy score for the lactic acid vaginal gel group was 2.07 at week three, 2.38 at month three, and 2.75 at month six, while the tolerability was 1.5, 2, and 2, respectively. At month three, 9 of 13 women (69.2%) would recommend the lactic acid vaginal gel to others.

## Discussion

Due to rising rates of antimicrobial resistant bacteria, finding non-antibiotic treatment options for acute BV is of increasing importance. Maintaining a low vaginal pH is key to supporting the concentration of lactobacilli and keeping BV-associated bacteria at bay [17]. In vitro studies have shown that the use of exogenous lactic acid can support the local vaginal flora and help eradicate BV-associated anaerobes [10]. This has yet to be conclusively proven in vivo [11]. This pilot study is consistent with preexisting research, showing inconclusive but promising results concerning the efficacy of lactic acid vaginal gel in the treatment of BV.

The Amsel criteria and Nugent score are the gold standards for diagnosing acute BV. 60% of patients receiving the lactic acid vaginal gel product were considered cured after three weeks based on the Amsel criteria and 40% based on the microscopically determined Nugent score and clue cells. This is consistent with documented cure rates of other lactic acid products with similar lactic acid concentrations and pH values of the intervention product, treatment regimens and follow-up times [11]. Compared to the standard antibiotic treatment however, these short-term cure rates are significantly inferior. Several factors could contribute to this difference. Firstly, objective outcomes were only measured at the start and after three weeks, and no objective improvements were observed from visit 1 to visit 2. This indicates that the lactic acid vaginal gel may not have produced significant changes in objective measures in the short term. There is no data to determine whether the lactic acid vaginal gel would have a slower, but more long-lasting positive effect on the vaginal flora. Future research should address this gap by including long-term follow-up assessments to evaluate the sustained efficacy of the treatment. Secondly, polymicrobial biofilms have been described as particularly resistant to lactic acid, which could further inhibit the effectiveness of the study product [18]. Moreover, the local concentration of lactic acid in the vaginal mucosa could not be measured, leaving uncertainty about whether a biologically efficient concentration was achieved despite proper application. Incorrect application is also more likely with vaginal applicators than with oral medication.

It is also noteworthy that only 50% of the patients included had a Nugent score consistent with BV, despite having three or more positive Amsel criteria. These inconsistencies have been described before. However, Andersch et al. report that the diagnosis of BV should be based more on clinical criteria rather than microbiological findings [19]. Also, the prevalence of *Gardnerella vaginalis* was reported as 65.6% between both groups, which is significantly less than previously described numbers in women with BV [20]. Both, the Amsel criteria and the Nugent score were determined in various institutions by a number of different clinicians, and there was no consistency between visits or inter-individually. This variability in diagnostic practices could have influenced the study results.

Strengths of this study include its focus on a non-antibiotic treatment option, which is highly relevant in the context of increasing antibiotic resistance. The lactic acid vaginal gel was well-tolerated by patients, and no significant safety concerns were reported. Additionally, the gel effectively reduced subjective symptoms such as discharge, malodor, and itching, performing comparably to metronidazole in these aspects.

However, several limitations should be noted. The main limitation of this study is the lack of masking, which could introduce bias. To address this issue in future research, we suggest considering a double-dummy design for larger trials to ensure that both participants and clinicians are masked to the treatment allocations. The short-term efficacy of the lactic acid vaginal gel was only assessed at the study’s start and after three weeks, leaving a gap in data regarding its long-term effects on the vaginal flora. Polymicrobial biofilms, which are resistant to lactic acid, may have also inhibited the product’s effectiveness. Furthermore, the local concentration of lactic acid in the vaginal mucosa could not be measured, casting doubt on whether a biologically effective concentration was achieved. Additionally, the study had a relatively low retention rate of 46.88% after six months, which could introduce attrition bias. Variability in diagnoses due to different clinicians and institutions determining the Amsel criteria and Nugent scores may have further impacted the results. The lack of a microbiological examination in the long-term follow-up and the small sample size also limit the study’s findings.

The clinical implications of this study are significant in the context of rising antibiotic resistance. The findings suggest that lactic acid vaginal gel could be a viable non-antibiotic treatment option for BV, particularly in cases where antibiotics are not suitable or where resistance is a concern. Although the short-term efficacy of the lactic acid gel was comparable to metronidazole in reducing subjective symptoms, its higher recurrence rate and the potential for variability in its effectiveness highlight the need for further research. This study contributes to the literature by providing preliminary evidence that supports the potential role of lactic acid in managing BV and underscores the need for larger, long-term studies to confirm these findings and refine treatment guidelines.

Both groups showed non-significant differences in recurrence rates after three and six months. The lactic acid vaginal gel group, however, had a recurrence rate of 37.5%, which was more than double that of the metronidazole group (14.3%). In the past, supplementing metronidazole with lactic acid vaginal gel has performed better in preventing both, objective and subjective recurrences of BV than metronidazole or lactic acid alone [21]. Therefore, combining antibiotics with a vaginal lactic acid gel could potentially offer the most effective strategy for treating BV and reducing recurrence rates.

Regarding subjective symptoms, the lactic acid vaginal gel performed equally as well as metronidazole and was successful in reducing discharge by 69%, vaginal malodor by 50% and itching by 84% within the first three weeks. By month six, all patients who did not currently have a BV-recurrence were free of symptoms. However, the lack of masking for both patients and clinicians raises the possibility that these symptom improvements might be influenced by the placebo effect.

For future studies, it could be beneficial to include a microbiological examination in the long-term follow-up to improve objectivity. Also, an extensive subgroup analysis was not possible in this case due to the small sample size. It would be of interest to examine the correlation between all individual patient characteristics and likelihood of cure, and if in clinical settings individual characteristics or symptoms should influence the decision for or against lactic acid vaginal gel as a treatment option. As rates of antibiotic resistant bacteria are rising, vaginal lactic acid products could prove to be an effective alternative first-line treatment for BV where antibiotics would be prescribed in severe cases or if symptoms persist.

## Conclusion

In conclusion, this pilot study aimed to compare the efficacy of a lactic acid vaginal gel to metronidazole, the standard treatment for BV. We conclude that: (1) lactic acid vaginal gel is a safe and generally well-tolerated alternative that is readily available without a prescription; (2) there is data to indicate that lactic acid vaginal gel can perform equally as well as metronidazole in certain outcomes, e.g. subjective symptom improvement and recurrence prevention, and (3) the efficacy of lactic acid vaginal gel should definitely be examined in a larger-scale study.

## Electronic Supplementary Material

Below is the link to the electronic supplementary material.

Supplementary Material 1

Supplementary Material 2: Case report form

## Data Availability

The datasets generated and/or analyzed during the current study are available from the corresponding author upon reasonable request.

## Abbreviations

BV: Bacterial vaginosis
e.g.: Exempli gratia (engl. “for example”)
HIV: Human immunodeficiency virus
NS: Nugent score
NSS: Nugent scoring system
OR: Odds ratio
PCR: Polymerase chain reaction
pH: Potential hydrogen
PID: Pelvic inflammatory disease
ROS: Reactive oxygen species
STDs: Sexually transmitted diseases
