# Supplementary material for: Effectiveness and tolerability of lactic acid vaginal gel compared to oral metronidazole in the treatment of acute symptomatic bacterial vaginosis: a multicenter, randomized-controlled, head-to-head pilot study
Source: BMC Womens Health. 2025 Jan 6;25:7. doi: 10.1186/s12905-024-03513-1 (PMC11702144; doi:10.1186/s12905-024-03513-1)
Supplement: Supplementary file 2 — Supplementary Material 2: Case report form [file 12905_2024_3513_MOESM2_ESM.docx]

Additional File 2: Case report form

Multicenter, randomized-controlled, head-to-head pilot study in non-pregnant women with acute symptomatic bacterial vaginosis

Fiona Tidbury^1^, M. D., Grégory Brülhart^2^, M. D., Gabriela Müller^2^, M. D., Susanna Weidlinger^1^, M. D., Gerrit Eichner^3^, Ph. D., Michael von Wolff^1^, M. D., Petra Stute^1^, M. D.

1 Department of Obstetrics and Gynecology, University Hospital of Bern, Switzerland

2 Place de la Gare 15, 1700 Fribourg, Switzerland

3 Mathematical Institute, Justus-Liebig University of Giessen, Giessen, Germany

Corresponding author

Professor Dr. med. Petra Stute, M. D.

Department of Obstetrics and Gynaecology

University Hospital Inselspital

Friedbuehlstrasse 19, 3010 Bern, Switzerland

E-mail: petra.stute@insel.ch

Telephone: (00)41-31-632-1303

Fax: (00)41-31-632-1305

ORCID: 0000-0002-5591-1552

| **CASE REPORT FORM** | Bacterial vaginosis |
| --- | --- |

| **Multicenter, randomized-controlled, head-to-head pilot study in non-pregnant women with acute symptomatic bacterial vaginosis**  PI: Prof Dr. med. Petra Stute |
| --- |

| Cantonal Ethics committee No.: |  |
| --- | --- |
| University Hospital No.: |  |

| Study No./Code: |  |
| --- | --- |
| Randomization group: |  |

**Physician:** (Stamp/Date/Signature)

**Patient No. Date Application**

**Date Treatment Start Date Treatment End Treatment Duration**

**Days**

**Application according to instructions ! yes**

| Inclusion criteria | | yes |  | no |
| --- | --- | --- | --- | --- |
| 1 | 18 years of age |  |  |  |
|  |  |  |  |  |
| 2 | Acute symptomatic bacterial vaginosis |  |  |  |
|  |  |  |  |  |
| 3 | Signed informed consent |  |  |  |
| **Exclusion criteria** | | yes |  | no |
|  | Insufficient knowledge of German |  |  |  |
| 1 |  |  |  |  |
|  |  |  |  |  |
| 2 | Illiteracy |  |  |  |
|  |  |  |  |  |
| 3 | Pregnancy (excluded using urine sample) |  |  |  |
|  |  |  |  |  |
| 4 | Other acute illness |  |  |  |
|  |  |  |  |  |
| 5 | Known allergy against any ingredients of the investigational products |  |  |  |

**VISIT 1**

| **DEMOGRAPHIC DATA**  Age (years):............ Height (m):............. Weight (kg):............... BMI (kg/m2):.............. |
| --- |

| **VITAL SIGNS**  Pulse (bpm):............. Blood pressure (mmHg):........................... Body temperature (°C):................. |
| --- |

| PHYSICAL EXAMINATION | | | |
| --- | --- | --- | --- |
| Code | System | *Abnormal | Normal |
| 1 | General condition |  |  |
| 2 | Cardial |  |  |
| 3 | Respiratory |  |  |
| 4 | Abdomen |  |  |
|  | | | |
| * If ***ABNORMAL*** please give details below. | | | |
|  | | | |
| Code | Details | | |
|  |  | | |
|  |  | | |
|  |  | | |

| **PATIENT HISTORY** | |
| --- | --- |
| Are you currently in a stable partnership? | ☐ No ☐ Yes |
| How many sexual partners in the past year? | Number: |
| Are you currently sexually active? | ☐ No ☐ Yes |
| How many vaginal symptoms in the past six months? | Number: |
| Do you smoke? | ☐ No ☐ Yes |
| Do you drink more than one unit per week of alcohol? | ☐ No ☐ Yes |
| Do you suffer from any gynecological illnesses? If yes, which? | ☐ No ☐ Yes  ___________________________ |
| Do you suffer from any metabolic illnesses? If yes, which? | ☐ No ☐ Yes  ___________________________ |
| Do you suffer from any dermatological illnesses? If yes, which? | ☐ No ☐ Yes ___________________________ |
| Do you have/Have you had cancer? If yes, which? | ☐ No ☐ Yes  ___________________________ |
| How many vaginal births have you had? | Number: |
| Contraceptive method | ☐ Natural family planning  ☐ combined hormonal contraception (pill, vaginal ring, plaster)  ☐ Progesterone contraception (pill, Implanon, injections)  ☐ condom  ☐ coil  ☐ sterilisation  ☐ diaphragm  ☐ other:_________________ |
| Do you regularly take medication? If yes, which? | ☐ No ☐ Yes ___________________________  ___________________________  ___________________________  ___________________________  ___________________________  ___________________________  ___________________________ |
| Do you have any known allergies? If yes, which? | ☐ No ☐ Yes ___________________________  ___________________________ |

| **URINE TEST STRIP** | |
| --- | --- |
| Pregnancy | ☐ Negative ☐ Positive |
| pH | _________ |
| Leucocytes | ☐ Negative ☐ Positive |
| Nitrite | ☐ Negative ☐ Positive |
| Protein | ☐ Negative ☐ Positive |
| Blood | ☐ Negative ☐ Positive |
| Glucose | ☐ Negative ☐ Positive |

| **AMSEL CRITERIA** | |
| --- | --- |
| Abnormal vaginal discharge | ☐ No ☐ Yes |
| Vaginal pH | _________ |
| KOH-Test | ☐ Negative ☐ Positive |
| Clue Cells | ☐ Negative ☐ Positive |
| Amsel Score | ............................... |

| **OTHER** | |
| --- | --- |
| General bacteriology | ☐ Negative ☐ Positive |
| Trichomonas vaginalis | ☐ Negative ☐ Positive |
| Mycoplasma hominis | ☐ Negative ☐ Positive |
| Neisseria gonorrhea | ☐ Negative ☐ Positive |
| Chlamydia trachomatis | ☐ Negative ☐ Positive |

| **SUBJECTIVE SYMPTOMS** | |
| --- | --- |
| Abnormal vaginal discharge | ☐ No (0 Points) ☐ Yes (1 Point) |
| Vaginal odor | ☐ No (0 Points) ☐ Yes (1 Point) |
| Vaginal pain | ☐ No (0 Points) ☐ Yes (1 Point) |
| Vaginal itching | ☐ No (0 Points) ☐ Yes (1 Point) |
| Burning sensation | ☐ No (0 Points) ☐ Yes (1 Point) |
| Vaginal dryness | ☐ No (0 Points) ☐ Yes (1 Point) |
| Subjective Symptom Score | ................. |

**VISIT 2**

| **AMSEL CRITERIA** | |
| --- | --- |
| Abnormal vaginal discharge | ☐ No ☐ Yes |
| Vaginal pH | _________ |
| KOH-Test | ☐ Negative ☐ Positive |
| Clue Cells | ☐ Negative ☐ Positive |
| Amsel Score | ............................... |

| **SUBJECTIVE SYMPTOMS** | |
| --- | --- |
| Abnormal vaginal discharge | ☐ No (0 Points) ☐ Yes (1 Point) |
| Vaginal odor | ☐ No (0 Points) ☐ Yes (1 Point) |
| Vaginal pain | ☐ No (0 Points) ☐ Yes (1 Point) |
| Vaginal itching | ☐ No (0 Points) ☐ Yes (1 Point) |
| Burning sensation | ☐ No (0 Points) ☐ Yes (1 Point) |
| Vaginal dryness | ☐ No (0 Points) ☐ Yes (1 Point) |
| Subjective Symptom Score | ................. |

FOR GYNOFIT®–GROUP:

| How do you perceive the efficacy of Gynofit®? | ☐ very good  ☐ good  ☐ satisfactory  ☐ unsatisfactory |
| --- | --- |
| How tolerable was Gynofit®? | ☐ very good  ☐ good  ☐ satisfactory  ☐ unsatisfactory |

| **COMPLIANCE** | |
| --- | --- |
| Returned empty blisters or applicators | (Gynofit group: 12 empty applicators  Metronidazole group: 14 empty blisters) |

| **ADVERSE EVENTS** | |
| --- | --- |
| Has the patient experienced any side effects since the signing of informed consent? If yes, please give details below. | ☐ No ☐ Yes |

| Adverse Events | | | | | | | | | | | | | | | | | | | | | | | | |
| --- | --- | --- | --- | --- | --- | --- | --- | --- | --- | --- | --- | --- | --- | --- | --- | --- | --- | --- | --- | --- | --- | --- | --- | --- |
|  | |  | | |  | | | | | | | | | | | | | | |  | |  | | |
| Has the patient experienced any Adverse Events since signing the Informed Consent? | | | | | | | | | | | |  | Yes, specify below | | | | | | |  | | No | | |
|  | | | | | | | | | | | | | | | | | | | | | | | | |
| **AE no.** | **Adverse Event** (diagnosis (if known) or signs/symptoms) | | **Start Date**  dd/mmm/yyyy  **and Time**  (24 hour clock) | **Stop Date**  dd/mmm/yyyy  **and Time**  (24 hour clock) | | **Outcome**  1=Recovered  2=Recovered with sequelae  3=Continuing  4=Patient Died  5=Change in AE  6=unknown | **Severity**  1=Mild  2=Moderate  3=Severe | **Plausible relationship to Study Drug** | | | **Action taken with Study Drug**  1=None  2=Dose Reduction Temporarily  3=Dose Reduced  4=Discontinued Temporarily  5=Discontinued | | | **Withdrawn due to AE?** | | | **Serious AE (SAE)?** | | | | **If SAE does it require immediate reporting?** | | | |
|  |  | |  |  | |  |  |  |  |  |  | | |  |  |  |  |  |  | |  | |  |  |
|  |  |  | / / | / / | |  |  |  |  | Yes |  |  |  |  |  | Yes |  |  | Yes | |  | |  | Yes |
|  |  |  |  |  | |  |  |  |  |  |  |  |  |  |  |  |  |  |  | |  | |  |  |
|  |  |  | **:** | **:** | |  |  |  |  | No |  |  |  |  |  | No |  |  | No | |  | |  | No |
|  |  |  |  |  | |  |  |  |  |  |  |  |  |  |  |  |  |  |  | |  | |  |  |
|  |  | |  |  | |  |  |  |  |  |  | | |  |  |  |  |  |  | |  | |  |  |
|  |  |  | / / | / / | |  |  |  |  | Yes |  |  |  |  |  | Yes |  |  | Yes | |  | |  | Yes |
|  |  |  |  |  | |  |  |  |  |  |  |  |  |  |  |  |  |  |  | |  | |  |  |
|  |  |  | **:** | **:** | |  |  |  |  | No |  |  |  |  |  | No |  |  | No | |  | |  | No |
|  |  |  |  |  | |  |  |  |  |  |  |  |  |  |  |  |  |  |  | |  | |  |  |
|  |  | |  |  | |  |  |  |  |  |  | | |  |  |  |  |  |  | |  | |  |  |
|  |  |  | / / | / / | |  |  |  |  | Yes |  |  |  |  |  | Yes |  |  | Yes | |  | |  | Yes |
|  |  |  |  |  | |  |  |  |  |  |  |  |  |  |  |  |  |  |  | |  | |  |  |
|  |  |  | **:** | **:** | |  |  |  |  | No |  |  |  |  |  | No |  |  | No | |  | |  | No |
|  |  |  |  | Studie Bakterielle Vaginose Randomisierungs-Nr.:  KEK-Nr.: Studienarm:  Insel-Nr.: | |  |  | Studie Bakterielle Vaginose Randomisierungs-Nr.:  KEK-Nr.: Studienarm:  Insel-Nr.: | Studie Bakterielle Vaginose Randomisierungs-Nr.:  KEK-Nr.: Studienarm:  Insel-Nr.: | Studie Bakterielle Vaginose Randomisierungs-Nr.:  KEK-Nr.: Studienarm:  Insel-Nr.: |  |  |  | Studie Bakterielle Vaginose Randomisierungs-Nr.:  KEK-Nr.: Studienarm:  Insel-Nr.: | Studie Bakterielle Vaginose Randomisierungs-Nr.:  KEK-Nr.: Studienarm:  Insel-Nr.: | Studie Bakterielle Vaginose Randomisierungs-Nr.:  KEK-Nr.: Studienarm:  Insel-Nr.: |  |  |  | |  | |  |  |

**VISIT 3 (Telephone Follow-up 1)**

| GENERAL HEALTH | |
| --- | --- |
| Have you had any new illnesses since the last visit? If yes, which? | ☐ No  ☐ Yes  ____________________________________ |
| Have you received antibiotic treatment since the last visit? If yes, why? | ☐ No  ☐ Yes  ____________________________________ |
| Have you taken any new medication since the last visit? If yes, which? | ☐ No  ☐ Yes  ____________________________________ |
| Are you pregnant? If yes, specify gestational age. | ☐ No  ☐ Yes  ____________________________________ |

| GYNECOLOGICAL FOLLOW-UP | |
| --- | --- |
| Have you been diagnosed with any of the following infections since the last visit? | ☐ Bacterial vaginosis  ☐ Chlamydia  ☐ Gonorrhea  ☐ Syphillis  ☐ Trichomoniasis  ☐ Herpes genitalis  ☐ Genital warts  ☐ Andere:________________________ |
| Do you have any of the following symptoms? | ☐ Vaginal discharge  ☐ Vaginal odor  ☐ Vaginal pain  ☐ Vaginal itching  ☐ Burning sensation  ☐ Vaginal dryness  ☐ No symptoms |
| Have you had unprotected sexual intercourse since the last visit? | ☐ Yes  ☐ No |
| Do you have a different sexual partner since the last visit? | ☐ Yes  ☐ No |
|  |  |
| For Gynofit®-Group | |
| How do you perceive the efficacy of Gynofit®? | ☐ very good  ☐ good  ☐ satisfactory  ☐ unsatisfactory |
| How tolerable was Gynofit®? | ☐ very good  ☐ good  ☐ satisfactory  ☐ unsatisfactory |
| Would you recommend Gynofit® to others? | ☐ Yes  ☐ No |

**VISIT 4 (Telephone Follow-up 2)**

| GENERAL HEALTH | |
| --- | --- |
| Have you had any new illnesses since the last visit? If yes, which? | ☐ No  ☐ Yes  ____________________________________ |
| Have you received antibiotic treatment since the last visit? If yes, why? | ☐ No  ☐ Yes  ____________________________________ |
| Have you taken any new medication since the last visit? If yes, which? | ☐ No  ☐ Yes  ____________________________________ |
| Are you pregnant? If yes, specify gestational age. | ☐ No  ☐ Yes  ____________________________________ |

| GYNECOLOGICAL FOLLOW-UP | |
| --- | --- |
| Have you been diagnosed with any of the following infections since the last visit? | ☐ Bacterial vaginosis  ☐ Chlamydia  ☐ Gonorrhea  ☐ Syphillis  ☐ Trichomoniasis  ☐ Herpes genitalis  ☐ Genital warts  ☐ Andere:________________________ |
| Do you have any of the following symptoms? | ☐ Vaginal discharge  ☐ Vaginal odor  ☐ Vaginal pain  ☐ Vaginal itching  ☐ Burning sensation  ☐ Vaginal dryness  ☐ No symptoms |
| Have you had unprotected sexual intercourse since the last visit? | ☐ Yes  ☐ No |
| Do you have a different sexual partner since the last visit? | ☐ Yes  ☐ No |
|  |  |
| For Gynofit®-Group | |
| How do you perceive the efficacy of Gynofit®? | ☐ very good  ☐ good  ☐ satisfactory  ☐ unsatisfactory |
| How tolerable was Gynofit®? | ☐ very good  ☐ good  ☐ satisfactory  ☐ unsatisfactory |
| Would you recommend Gynofit® to others? | ☐ Yes  ☐ No |
